# Evolution of Fructans in *Aguamiel* (Agave Sap) During the Plant Production Lifetime

**DOI:** 10.3389/fnut.2020.566950

**Published:** 2020-10-08

**Authors:** Ibeth Peralta-García, Fernando González-Muñoz, Rodríguez-Alegría María Elena, Alejandro Sánchez-Flores, Agustín López Munguía

**Affiliations:** ^1^Departamento de Ingeniería Celular y Biocatálisis, Instituto de Biotecnología, Universidad Nacional Autónoma de México (UNAM), Cuernavaca, Mexico; ^2^Unidad de Secuenciación Masiva y Bioinformática, Instituto de Biotecnología, Universidad Nacional Autónoma de México, Cuernavaca, Mexico

**Keywords:** *aguamiel*, *metzal*, *pulque*, scraped, pine, fructans, fructo-oligosaccharides, sucrose

## Abstract

*Aguamiel* is the sap collected from *agave*, while *pulque* is the result of the natural fermentation of *aguamiel*. Despite its ancestral origin and numerous publications on *pulque* production, little is known about the evolution and concentration of sugars and fructo-oligosaccharides in *aguamiel*, either during its daily accumulation or through the agave production lifetime. In this study, we examined *aguamiel* composition in three agave plants during their productive lifetime (4 to 9 months). After each collection, the agave pine is scraped to induce *aguamiel* to flow into an internally created cavity (*cajete*), producing a residual bagasse (*metzal*). We found that the concentration of agave fructans and sucrose, as well as the fructan profile, change during the *aguamiel* production process. During the daily collection, a small amount of agave fructans released from the pine by scraping is drawn into the *cajete* with the first milliliters of sap where it is then diluted with the inflow of *aguamiel*. The main component of *aguamiel* is the sucrose produced in high concentration in the leaves through photosynthesis and then hydrolyzed in the *cajete* as *aguamiel* accumulates. We also describe how the fructan profile changes during the accumulation of *aguamiel* in the *cajete*. In addition to the varying amount of sucrose that is hydrolyzed in the *aguamiel* accumulated, we found that fructo-oligosaccharides are either diluted, consumed, or hydrolyzed, depending on the plant and its production stage, thus yielding different fructan profiles. New fructo-oligosaccharides are, in some cases, synthesized by bacteria present in *aguamiel*. These profiles were also observed in *aguamiel* collected from ten different plants in the same production region. We also found that a considerable amount of agave fructans is lost in *metzal* (bagasse), the agave material that is scraped and thrown away twice a day during the production process.

## Introduction

The genus *Agave* is endemic to the American continent. Some 75% of the known *Agave* species occur in Mexico (1), where several of them are used to produce traditional distilled alcoholic beverages, such as *mezcal, tequila, bacanora, sotol* … (2). *Pulque* is the only non-distilled alcoholic beverage derived from the fermentation of agave sap, as recently reviewed by Escalante et al. (3).

*Aguamiel* is a colorless, sweet sap obtained from agave species such as *A. salmiana, A. mapisaga, A. americana, A. atrovirens, A. hookerii, A*. *inaequidens*, and *A. marmorata* (4) which, after spontaneous fermentation, yields the beverage known as *pulque*. This beverage has interesting nutritional properties, as it contains essential amino acids, minerals such as K, Ca, Na, Fe, Cu, Mg, Se, and Zn; vitamin B complex and vitamin C, antioxidants (saponins and phenolic compounds), soluble fiber consisting of fructans from the plant and/or glucans produced by lactic acid bacteria, as well as probiotics (5–7). Fructans are fructose polymers that are indigestible by human gastrointestinal enzymes but that selectively stimulate beneficial gut microorganisms such as *Bifidobacteria* and *Lactobacillus* among others (8).

Numerous scientific studies have demonstrated that the effect of fructan consumption on human health goes beyond their prebiotic activity, as they also contribute to reduce oxygen reactive species, promote calcium reabsorption, and are associated with a decrease in serum triglyceride and cholesterol concentrations (9). Research on animal models has also demonstrated that a fructan-supplemented diet regulates the beneficial microbiota and decreases brain damage and deterioration (10). All these properties may explain the health benefits of *pulque* consumption in Mexican rural communities.

With regard to the agave industry, it is important to consider that, unlike *tequila, mezcal*, and other distilled beverages that require fructans hydrolysis by thermal treatment, *pulque* fermentation is based on the fermentable sugars of *aguamiel*, mostly sucrose, thus preserving agave fructans (11). After centuries of traditional fermentation and decades of scientific research, the changes in concentration and composition (molecular weight distribution) of fructans in *aguamiel* have not been yet studied, particularly as regards the changes that take place during the agave productive lifetime. Sugar concentration in accumulated *aguamiel* is routinely reported; however, the changes in *aguamiel* composition during production after scraping and at different production stages have never been reported (12, 13).

*Aguamiel* production starts when the agave plant reaches maturity (8 to 10 years) and immediately before flowering. At that time, the producer (*tlachiquero*) performs a process known as “castration” or “layering,” consisting of removing the central leaves of the plant and carving a cavity (traditionally known as *cajete*) to remove the apical meristem (*cogollo* or heart), which otherwise would form the inflorescence. Sap flows and accumulates in the *cajete* (3, 14), which is scraped twice a day after collecting the *aguamiel* accumulated, while the scraped material (*metzal)* is discarded. The castration process varies from region to region: in Huitzilac, a town in the State of Morelos, where this study was conducted, the central cavity is punctured without removing the central leaves, only extracting from the *cogollo*, the center of the base.

After castration, the agave is left to mature for up to 3 months or even longer, depending on the region and the manufacturing practice (3). Producers in Huizilac leave the castrated agave to mature for 5 weeks before starting the scraping routine and the daily collection of *aguamiel*. For this purpose, the sides of the stem are scraped with a metal spoon (*ocaxtle)* specifically designed for the process, inducing sap to flow into the *cajete. Aguamiel* is collected once the *cajete* is full, after 12–14 h of accumulation. This procedure is carried out twice a day over 4 to 9 months, depending on the plant size, until the plant dies.

The accumulated *aguamiel* is traditionally extracted from the *cajete* using a sort of large straw made from a dried pumpkin (*acocote)*, but modern production processes use pumps. Afterwards, *aguamiel* is transported to the fermentation room (*tinacal*), where fermentation takes place after inoculation with freshly produced *pulque*. The whole process has been recently reviewed by Escalante et al. (15).

The sugars profile of *aguamiel* from several *Agave* species has been the subject of various studies (5, 7, 12, 13). However, no detailed information is yet available on the relationship between *aguamiel* composition and the plant productive stage, nor on changes in *aguamiel* composition during post-scraping accumulation. Ortiz-Basurto et al. (5) described changes in *aguamiel* composition during the agave productive lifetime, concluding that its components profile varies little over time. However, as described in this manuscript, their sampling procedures cannot be compared.

We hereby describe the evolution of *aguamiel* composition in three *pulque*-producing agave plants, in terms of the concentration of glucose, fructose, sucrose, and the concentration and molecular weight distribution of fructans. We also describe the changes occurring during *aguamiel* accumulation in the *cajete* and through the entire productive lifetime. We carried out an overall quantification of the process in each *Agave* plant to identify the fate of fructans, in either *aguamiel* or *metzal*, and to quantify the total amount of sucrose produced. A fourth plant was analyzed as a reference to describe the initial concentration and molecular weight profile of fructans in a pine prior to production. We also analyzed the influence of the microbiota in *aguamiel*. This is the first time that this ancestral process is thoroughly examined and described, providing important conclusions that may affect the way the extraction procedure evolves in order to improve the nutritional quality of the product and the control of the fermentation process.

## Materials and Methods

Agave plants were purchased from Mr. Salvador Cueto (“*Don Chava*”), a traditional *aguamiel* collector and *pulque* producer in Huizilac, State of Morelos, a locality some 15 km north of the state capital, Cuernavaca. Two of the plants (P1 and P2) were identified as *Agave mapisaga* and the third (P3) as *A. salmiana*, based on phenotypic features. An additional *A. salmiana* plant (P4) was used to quantify and identify the location of fructans in the pine. We thank Dr. Abisai J. García, from the Instituto de Biología, UNAM, for the taxonomic identification of the specimens.

### *Aguamiel* Sampling and Storage

Samples of *aguamiel* and scraped tissue (*metzal*) were collected, using sterile instruments, from the agave plants weekly for the first month and every other week thereafter, from the first extraction and during the entire plant productive lifetime. Samples were stored in a plastic container filled with solid CO_2_ almost immediately after collection, Once in the laboratory, *aguamiel* and *metzal* samples were stored at −20°C and −80°C, respectively, until analysis.

#### Changes in Composition During Aguamiel Extraction

Unless otherwise stated, 10–15 mL samples of fresh *aguamiel* were collected from the agave plants both immediately and at different times after scraping. On each occasion, the *cajete* was emptied prior to collecting the sample to avoid dilution. Two additional plants (P5 and P6) of the region were selected to monitor changes in *aguamiel* composition over time. Samples of accumulated *aguamiel* were collected from the *cajete* at the end of the 12–h period of daily accumulation.

#### Samples From a Reference *A. Salmiana* Plant

A mature agave plant (P4) was analyzed after defoliation (*jima*) to determine the natural distribution of sugars. The resulting leafless stem (commonly known as *piña* or pine) was taken to the *Instituto de Biotecnolog*í*a* of the UNAM (IBT-UNAM), where it was cut into six horizontal 4.5 cm-thick sections (see description in **Figure 7**): the first two and part of the third top sections are located at the base of the meristem and are followed by four additional sections down the stem. The stem was separated from the base of the leaves and samples collected, including samples of the tissue found at the leaf bases. A detailed description of the plant and the sections is provided in the Supplementary Material.

### Sample Preparation

#### Aguamiel Samples

*Aguamiel* samples were defrosted at 4°C, centrifuged at 14,000 g for 15 min, and filtered through 0.2 μm nylon syringe filters (Cronus 4 mm) to eliminate bacteria.

#### Sugar Extraction From Scraped and Pine Tissues

We used the following procedure to extract sugars from the scraped and pine tissues. A 5 g tissue sample was cut into 0.5 × 0.5 cm pieces and mixed with 10 mL of distilled water in a Hamilton Beach immersion mixer for 1 min (30 s at each speed setting). The mixture was centrifuged twice at 8 500 g for 15 min and filtered through 0.2 μm nylon syringe filters (Cronus 4 mm).

### Chemical Characterization of *Aguamiel* and *Scraped* and *Pine* Tissues

#### Identification of Simple Sugars

Simple sugars (fructose, glucose, and sucrose) were analyzed in a UltiMate^TM^ 166 3000 HPLC system equipped with an autosampler and a Shodex refractive index detector. Sugars in *aguamiel* were measured in a Gold Amino column (Thermo scientific, USA) kept at 30°C with acetonitrile (J.T. Baker®)/water (75:25, v/v) as mobile phase at 1.2 mL/min. Sugars in scraped tissue were measured with an Aminex-P column (Bio- Rad, USA), using water at 0.6 mL/min and 60°C as eluent to improve sucrose resolution and avoid the overlap with another sugar, probably stachyose, not present in *aguamiel*.

#### Fructan Characterization

Fructans concentration was measured by gel permeation chromatography (GPC) using a HPLC equipped with a linear Ultrahydrogel column (Waters, Japan) using running conditions described by Porras-Dominguez et al. (16), 0.8 mL/min of 0.1 mM NaNO_3_ at 30°C as eluent. Fructans profile was determined by HPAEC-PAD, using an ED50 electrochemical detector (Dionex, USA) and a CarboPac® PA200 column for carbohydrate analysis (Thermo scientific, USA) using running conditions stablished by Mellado-Mojica and López (17). Product elution was carried out by applying a sodium acetate gradient with 100 mM NaOH at 0.5 mL min^−1^ as follows: 5–100 mM sodium acetate in 25 min, 100–400 mM in 60 min, and 10 min for initial condition re-equilibration (5 mM sodium acetate) at 30°C. We determined the *number average molar mass* (Mn), *mass average molar mass* (Mw), and polydispersity index (PI) for each sample, as described by Porras-Dominguez et al. (16). From these data we estimated the degree of polymerization by number (**DPn)** and the degree of polymerization by mass (**DPw**) using linear regression analyses with data for glucose, sucrose, 1-ketose, nystose, fructosyl-nystose, as well as 5.2, 11.6, 23.8, and 48.6 KDa dextran as standards.

### Identification of Oligosaccharides in Accumulated *Aguamiel*

#### Identification of Dextran in Accumulated *Aguamiel*

Dextran and isomalto-oligosaccharides were identified by its selective enzymatic degradation in reactions with dextranase carried out according to Torres-Rodríguez et al. (18), with some modifications (300 U/L- Amano in 50 mM, 5.5 pH acetate buffer). The reaction was carried out at 60°C and 350 rpm for 4 h. A 2% w/v 5,200 Da dextran solution was used as reference, HPAEC-PAD profiles were compared.

#### Identification of Fructo-Oligosaccharides in Accumulated *Aguamiel*

Fructo-oligosaccharides (FOS) were identified by their selective enzymatic degradation with Fructozyme (Novozymes Corp., Denmark) the reaction was carried out as already described Torres-Rodríguez et al. (18), with some modifications (300 U/mL of enzyme and 50 mM, pH 5.5 acetate buffer solution). The reaction was performed at 40°C and 350 rpm for 15 h. A 1% concentration for aguamiel samples and for solution of *Preventy* a commercial form of *agave inulin* was used as reference.

### Statistical Analyses

All samples were analyzed in duplicate. Results are expressed as average plus standard deviation. ANOVAs were carried out using the software Minitab® version 18. Differences were considered significative when *p* < 0.05.

### *Aguamiel* Microbiota: DNA Extraction, Library Preparation, Sequencing, and Bioinformatic Analyses

A sample of fresh *aguamiel* was collected during winter. After centrifugation (9,000 g, 15 min at 4°C), the pellet containing the *aguamiel* biomass was washed with saline solution and the DNA extracted with the UltraClean Microbial DNA (MO BIO, QUIAGEN) commercial extraction kit, following the manufacturer instructions. Three milligrams of *aguamiel* total DNA with a 1.91 OD ratio (260 nm/280 nm) were obtained.

A sequencing library was prepared from total DNA from fresh *aguamiel* using the Illumina TruSeq DNA kit (Illumina, USA) following the manufacturer specifications with an average fragment size of 200–400 bp. The sequencing was performed on the NextSeq 500 (Illumina, USA) platform with a paired-end 75-cycle configuration at the *Unidad Universitaria de Secuenciación Masiva y Bioinformática* (UUSMB, for its acronym in Spanish) of the *Instituto de Biotecnolog*í*a*, UNAM, Mexico.

Quality of the reads was controlled, with previous adapter trimming and removal, using the software FastQC (http://www.bioinformatics.babraham.ac.uk). Taxonomic profiling of the microbial community was carried out with the program MetaPhlAn v2.7.7, based on a reference database of unique marker genes supplied with the program, using the default parameters. The taxa count table obtained was used to estimate the sampling effort and compute diversity indices with the packages Vegan v2.3.0 and phyloseq v1.12.2, respectively, in the Bioconductor R package set.

## Results and Discussion

### Daily Changes in *Aguamiel* Composition

We describe the composition of *aguamiel* collected either immediately after being exuded (fresh *aguamiel*) or, as commonly reported in the *aguamiel* literature, after its daily *accumulation* in the plant. To our knowledge, this is the first time that this approach is used in *aguamiel* research.

Sucrose was the only simple sugar found in fresh *aguamiel*. In contrast to previous reports [e.g., (5, 7)] for accumulated *aguamiel*, no other simple sugar (i.e., glucose or fructose) was found in fresh *aguamiel*. Enríquez et al. (13) reported that fructose and glucose concentration vary seasonally. We found (see below) these sugars only in accumulated *aguamiel*, after sucrose had been inverted either by endogenous or microbial enzymes.

**Figure 1:**
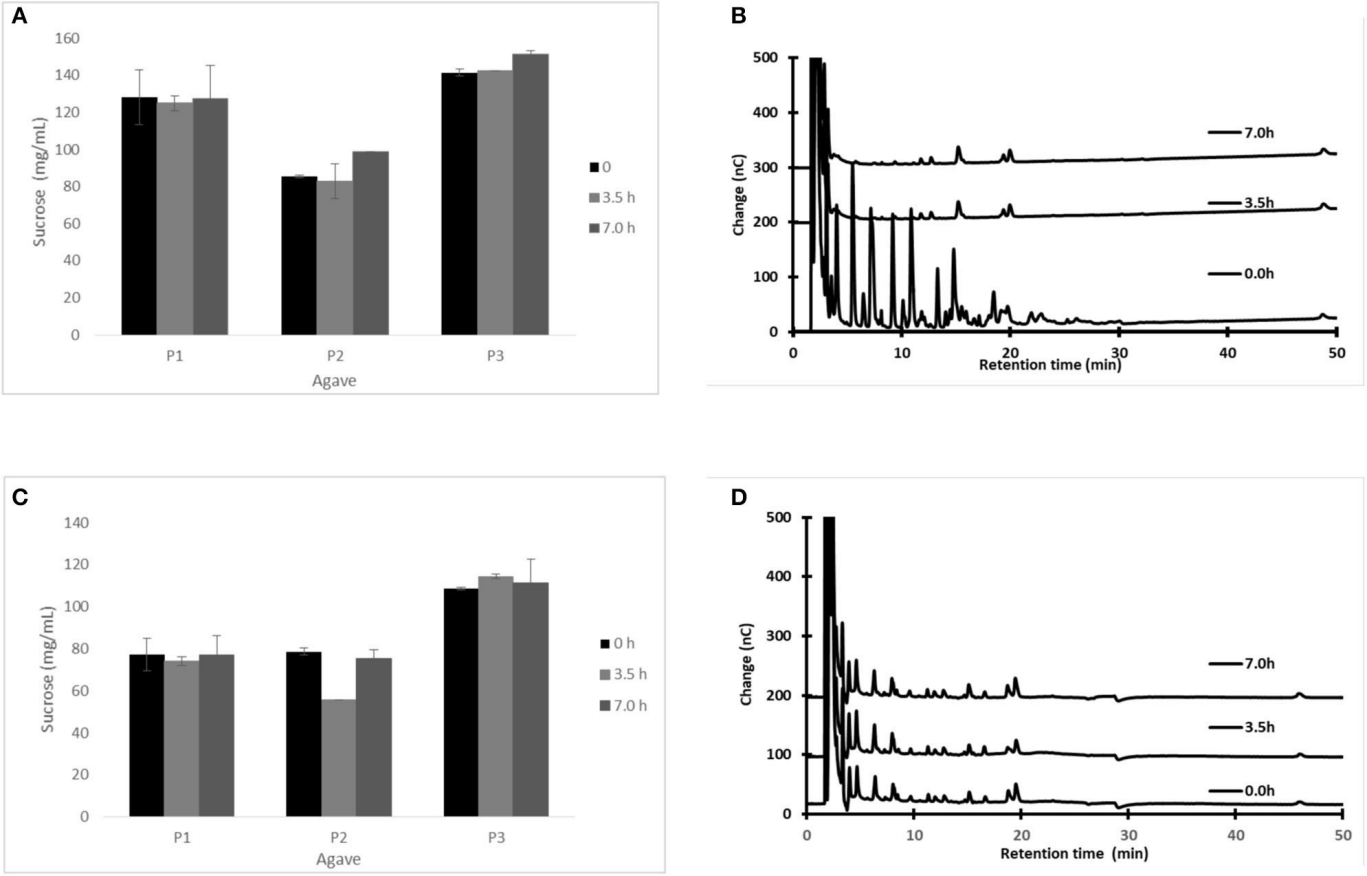
Sugar content in *aguamiel* at different collection times (0, 3.5, and 7 h after scraping) during a single day collection: sucrose **(A,C)** and fructo-oligosaccharide profile **(B,D)**. Scraping as usual before collection **(A,B)** or scraping before each *aguamiel* sample was collected **(C,D)**. Sucrose was measured by HPLC; FOS was measured by HPAEC-PAD. The HPAEC-PAD chromatograms **(B,D)** for plant P2 are shown as an example. Statistical differences: *p*-value for sucrose concentration in the traditional process for the three plants (P1, *p* = 0.968; P2, *p* = 0.111; and P3, *p* = 0.012). When the plant was scraped before sampling (P1, *p* = 0.779; P2, *p* = 0.005; and P3, *p* = 0.73). The *cajete* was previously emptied before sampling to avoid dilution.

Figure 1 shows the changes in sugar and fructan concentration in fresh *aguamiel* during 7 h on a production day, following the traditional scraping procedure (Figures 1A,B). We repeated the experiment, but scraping before collecting each *aguamiel* sample, as if the collection process had started again (Figures 1C,D). Figures 1A,C show that sucrose concentration remained almost constant throughout the day, independently of the scraping.

Surprisingly, in all cases fructans were found in high concentration in the first sample immediately after scraping (illustrated in Figures 1B,D at 0 h only for one of the three studied plants. However, as *aguamiel* flows, fructans in fresh *aguamiel* decrease (Figure 1B), suggesting that scraping releases fructans from the pine tissue, but these are washed out afterwards by the *aguamiel* flow. It has always been suggested that scraping stimulates *aguamiel* flow, but this is the first evidence demonstrating that scraping is the source of fructans in *aguamiel*.

As a result, while sucrose concentration in fresh *aguamiel* remains constant along the day, fructan concentration is high in *aguamiel* immediately after scraping but decreases as the sap flows (Figure 1B) and dilutes the fructans accumulated in the *cajete*. We conclude that scraping the *cajete* wall disrupts the surface tissue and the sap flow drags the fructans released from the apoplast along with sucrose from the phloem. In fact, our experiment shows that scraping the plant releases fructans any time (see Figure 1D).

The fact that fructans are found not only in the vacuole, but also in the apoplast (19), supports our explanation above. We can also speculate that fructans are mainly drained from inter-cellular spaces rather than from vacuoles, due to the difficulty of removing them from this organelle, where they are protected by the cell wall, the plasmatic membrane, and the tonoplast. This finding could have important consequences given the nutritional role of fructans, and the fate of the fructans remaining in the plant. Using alternative collection procedures could yield a higher fructan content in *aguamiel*, improving its soluble fiber content.

#### Fructan Dilution in *Aguamiel*

**Figure 2:**
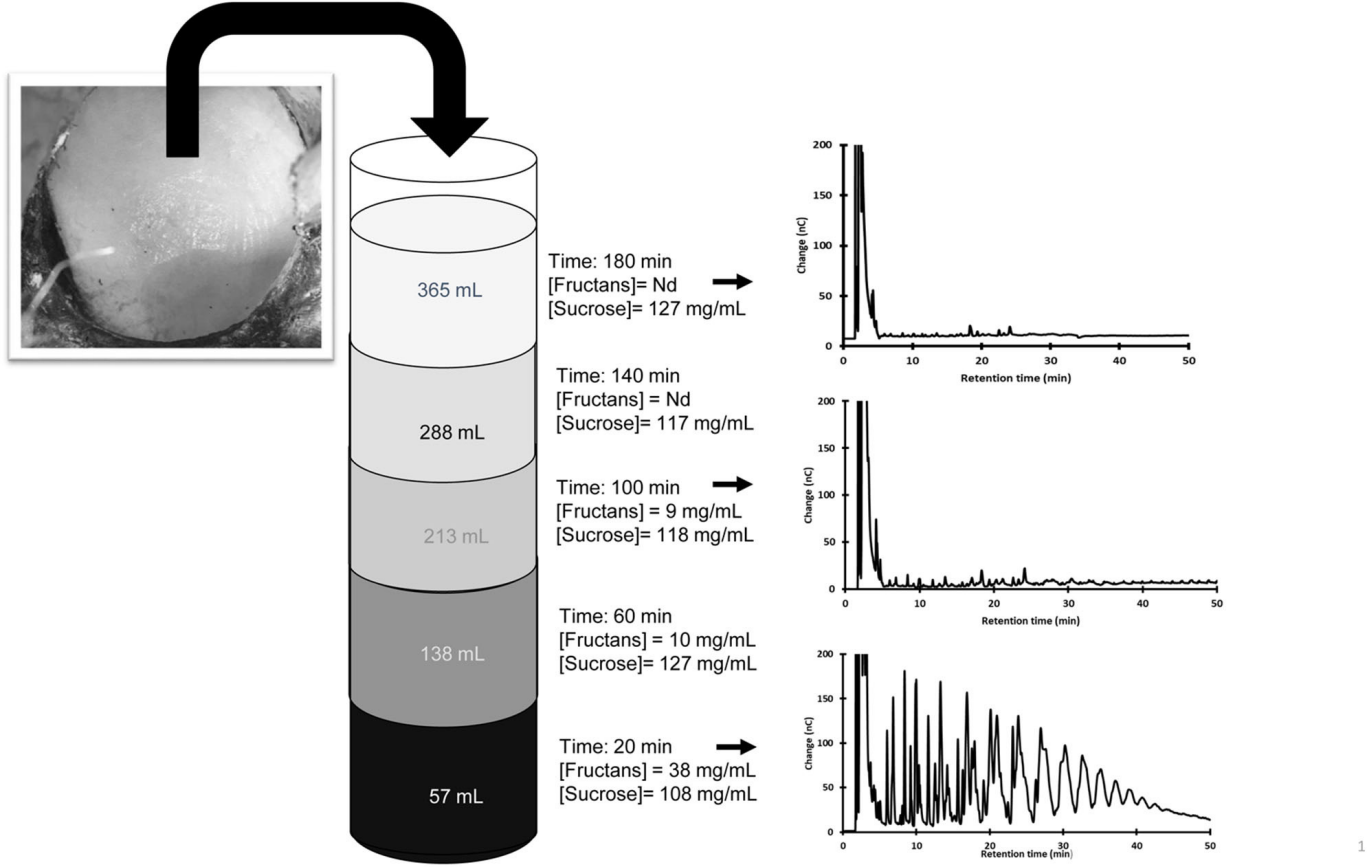
Changes in the concentration of sucrose, fructans, and the fructan profile in *aguamiel* exuded during the 3 h following scraping. *Aguamiel* samples were always collected in a previously emptied *cajete* to avoid dilution with accumulated *aguamiel*. Only three HPAEC profiles are shown.

The fructan dilution process in *aguamiel* was further studied in two additional plants (P5 and P6) growing in the same region. Fructans and simple sugars were measured in fresh *aguamiel* each 20 min after scraping. Figure 2 shows that 3 h after scraping fresh *aguamiel* no longer contains fructans, and therefore dilute the *aguamiel* accumulated in the *cajete*. This is referred in the next section as Type 3 *aguamiel*. Although the two agave plants were in the same production stage, they differed in their volume of *aguamiel* and fructan concentration; however, in both cases fructan concentration in fresh *aguamiel* became negligible after the flow of 200 mL. In contrast, sucrose concentration remained constant (*p*=0.533 and 0.937 for P5 and P6, respectively), throughout the sampling process confirming the previous observations.

#### Changes in *Aguamiel* Composition During Accumulation

A comparison of the carbohydrate profile of *aguamiel* collected after scraping and accumulated *aguamiel* in the three studied plants gave unexpected results. In all cases, a certain amount of sucrose was hydrolyzed during accumulation in the *cajete*, whereas the FOS profile varied between agave plants and their production stage (Table 1). This helps to explain the origin of the inverted sugar commonly found in *aguamiel*, as well as the significant variations in fructan concentration in *aguamiel* reported in the literature (5, 13, 20, 21).

**Table 1:** Changes in the concentration of simple sugars in accumulated and fresh *aguamiel* during the agave productive lifetime.

**Agave**	**Productive lifecycle (weeks)**		**pH**	**Fructose** **(mg/mL)**	**Glucose** **(mg/mL)**	**Sucrose** **(mg/mL)**	
		**Fresh**	**Accumulated**	**Accumulated**	**Accumulated**	**Fresh**	**Accumulated**
	5	9	3.5	34.3 ± 0.4	25.7 ± 0.1	85.2 ± 14.5	9.6 ± 0.1
P1	7	8.5	ND	9.2 ± 0.2	6.7 ± 0.6	141.1 ± 21.1	69.3 ± 3.7
	9	7	ND	10.0 ± 0.1	8.4 ± 0.1	116.2 ± 10.8	114.0 ± 0.1
	11	7	ND	10.1 ± 0.1	8.4 ± 0.1	99.8 ± 3.3	114.0 ± 0.1
	13	10	ND	5.2 ± 0.1	4.3 ± 0.1	127.8 ± 3.1	126.1 ± 0.1
P2	2	7	5	12.9 ± 0.1	8.8 ± 0.2	138.6 ± 4.0	18.9 ± 0.1
	3	7	5	57.5 ± 0.6	40.1 ± 0.4	163.5 ± 1.1	49.5 ± 4.7
	5	7	5	18.1 ± 0.1	63.5 ± 0.1	120.1 ± 4.6	98.8 ± 5.2
	7	7	5	41.7 ± 0.1	63.0 ± 0.9	119.6 ± 4.6	60.1 ± 2.9
	9	8	ND	8.8 ± 0.2	6.7 ± 0.3	98.8 ± 5.2	88.2 ± 2.5
P3	3	7.5	4.5	14.1 ± 0.1	13.2 ± 0.1	134.4 ± 6.0	87.7 ± 0.1
	5	7.5	4.5	18.7 ± 1.0	16.3 ± 0.1	130.1 ± 9.2	53.9 ± 0.1
	7	7	5	17.1 ± 0.1	14.8 ± 0.2	126.8 ± 10.5	89.1 ± 2.1
	9	7	5	18.0 ± 0.1	13.8 ± 0.1	86.2 ± 9.5	88.1 ± 0.1
	11	7	5	10.2 ± 0.1	6.6 ± 0.1	108.9 ± 5.0	94.3 ± 0.1

*Glucose and fructose were undetectable in fresh aguamiel (first 15–20 mL after scraping). ND, Not determined*.

*P1 and P2: A. mapisaga, P3: A. salmiana*.

**Figure 3:**
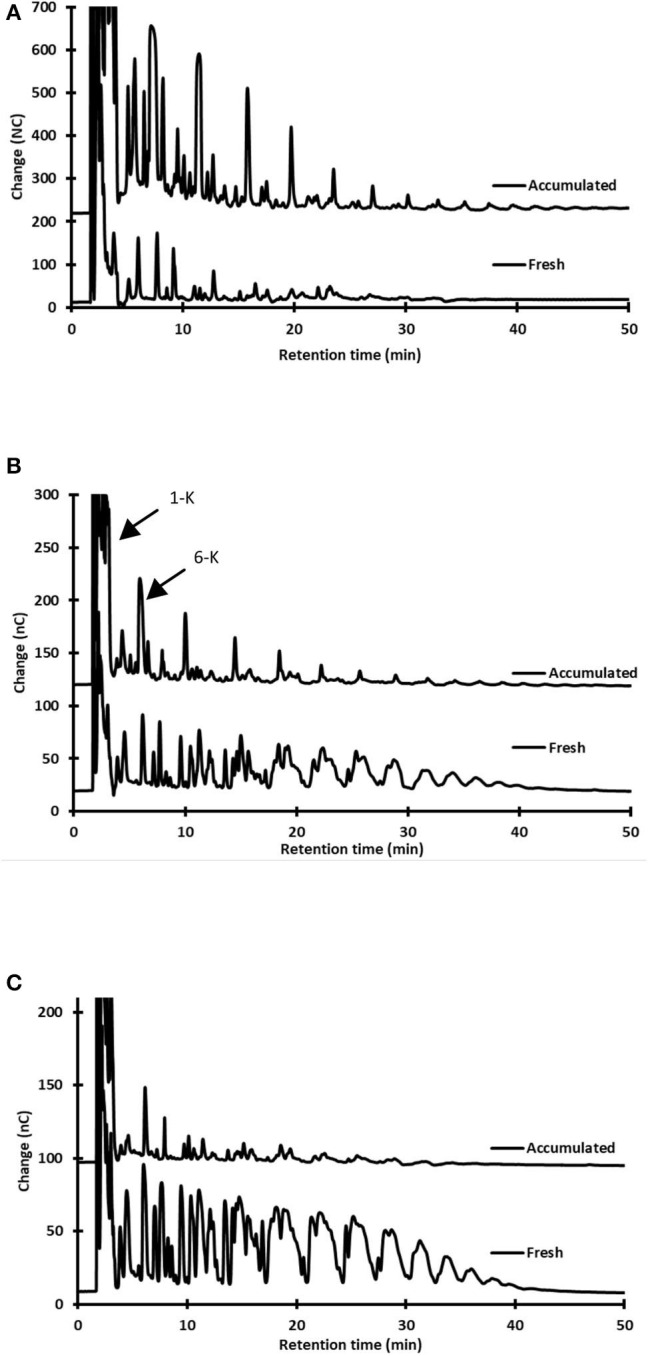
Examples of the three different FOS profiles observed in accumulated *aguamiel*. HPAEC-PAD oligosaccharide profiles in fresh (immediately after scraping) and accumulated (10 h) *aguamiel*. **(A) Type 1**. Synthesis of oligosaccharides: *A. mapisaga* in the early productive stage **(B) Type 2**. Agavin-like profile and synthesis of new oligosaccharides: *A. mapisaga* in intermediate-late productive stages, and **(C) Type 3**. Fructan dilution, *A. mapisaga* in an intermediate-late productive stage. See explanation in the text.

Figure 3 shows select chromatographs illustrating the differences in sugar content between *aguamiel* collected after scraping and accumulated *aguamiel*. In addition to the sucrose hydrolysis and the consequent presence of glucose and fructose (Table 1), the analysis showed three major differences (Types) of oligosaccharide (OS) profile:

**Type 1**. This carbohydrate profile was observed in the three studied plants during the early production stages. It is characterized by the synthesis of new oligosaccharides (OS), as concluded from the profiles in HPLC chromatograms. These OS can only result from the glycosyltransferase-mediated synthesis, which has been frequently reported in agave endogenous microorganisms and in those responsible of *pulque* production [(22) see also section Microbial Influence on Aguamiel Changes during Accumulation below]. These OS seem to have an inulin- or isomalto- oligosaccharide-type profile, quite different from the agave fructo-oligosaccharides (FOS). We may also conclude that fructans in this type of *aguamiel* are partially—and in some cases, almost completely—hydrolyzed or consumed by the *aguamiel* microbiota (Figure 3A). Manipulation of the plant since castration, as well as the harvesting procedure, introduce microorganisms, particularly those present in the tools used for scraping and extraction. In addition, endophytic bacteria may also contribute to this initial, *in-situ* transformation process of *aguamiel*, which will eventually render *pulque*. The *aguamiel* microbiota is analyzed in section Microbial Influence on Aguamiel Changes during Accumulation.
**Type 2**. This OS profile was mainly observed in *aguamiel* harvested from plants at intermediate and late production stages. It consists of a complex mixture of oligosaccharides showing a general agave-fructan profile, but with additional carbohydrate signals. Some agavin-type FOS signals in the HPLC chromatograms were higher than in *aguamiel* collected after scraping (Figure 3B). Two of these signals correspond to 1-kestose and 6-kestose that may result from agavin hydrolysis (23) or from the synthesis mediated by fructosyl transferase, considering that these trisaccharides are the earliest intermediates of inulin or levan synthesis, respectively (24, 25).
**Type 3**. The third type of OS profile was also found in the intermediate and late production stages, and simply corresponds to the dilution of the *aguamiel* fructans obtained after scraping. That is, the profile observed in *aguamiel* after scraping is diluted with the fructan-free sap as it accumulates in the *cajete* (Figure 3C). Only minor changes in pH and limited sucrose hydrolysis were observed in the type 3 profile. We suggest that this profile corresponds to *aguamiel* collected under the best hygiene conditions and would yield the highest quality substrate for *pulque*. We propose this profile to be considered as the baseline for quality control purposes if a reproducible composition substrate for *pulque* is intended. Although changes in composition are not necessarily detrimental, they depend on the *aguamiel* microbiota. Samples of accumulated *aguamiel* that showed major changes in fructan profile and sucrose hydrolysis also had the lowest pH as a result of a more intense microbial activity (Table 1).

To confirm these observations, samples of accumulated *aguamiel* were taken at random from ten agave plants growing in the Huitzilac region. The sample included different agave species and different production stages. Table 2 shows that, beyond the varying proportions of hydrolyzed sucrose, the ten samples consistently showed one of the three oligosaccharide profile types described above (see also the Supplementary Material).

**Table 2:** Classification of *aguamiel* obtained from 10 different agave plants from the Huitzilac region, based on the oligosaccharide profile.

**Agave**	**Productive stage**	**Species**	**Type of accumulated aguamiel**
A	Initial	*A. salmiana*	Type 1
B	Intermediate	*A. americana*	Type 3
B1	Intermediate	ND	Type 2
C	Final	*A. mapisaga*	Type 3
D	Intermediate	ND	Type 2
E	Intermediate	*A. salmiana*	Type 2
F	Final	ND	Type 2
G	Final	ND	Type 2
H	Initial	*A. salmiana*	Type 1
I	Intermediate	*A. americana*	Type 2

*ND, Not determined (uncertain)*.

Further studies might differentiate the quality and organoleptic properties of *pulque* obtained from these three types of *aguamiel*. Besides the plant condition through the different production stages we think that microorganisms play a major role in the accumulated aguamiel sugar profile. For instance, Valadez-Blanco et al. (26) demonstrate changes in *Zymomonas* influence during the first weeks of *aguamiel* production, as may be the case for other important microorganisms such as *Leuconostoc* species.

#### Identification of Oligosaccharides in Accumulated *Aguamiel*

We carried out two different analyses based on the specificity of hydrolytic enzymes to determine whether the OS found in type-1 and type-2 *aguamiel* are either fructose or glucose polymers. For the first analysis, samples of accumulated *aguamiel* were subjected to dextranase treatment, as several lactic acid bacteria (e.g., *Leuconostoc mesenteroides, L. dextranicum, L. citreum*, etc.) capable of synthesizing dextrans or isomalto-oligosaccharides from sucrose through glucosyltransferases have been described as agave endophytes (22, 27). Based on a similar hypothesis, for the second analysis the *aguamiel* samples were treated with Fructozyme, a combination of endo- and exo-fructanases able to hydrolyze fructans such as inulin or levan. The accumulation of either glucose or fructose, respectively, following these enzymatic reactions, along with the reduction of their HPLC signals, would indicate the synthesis of gluco- or fructo-oligosaccharides during *aguamiel* accumulation.

**Figure 4:**
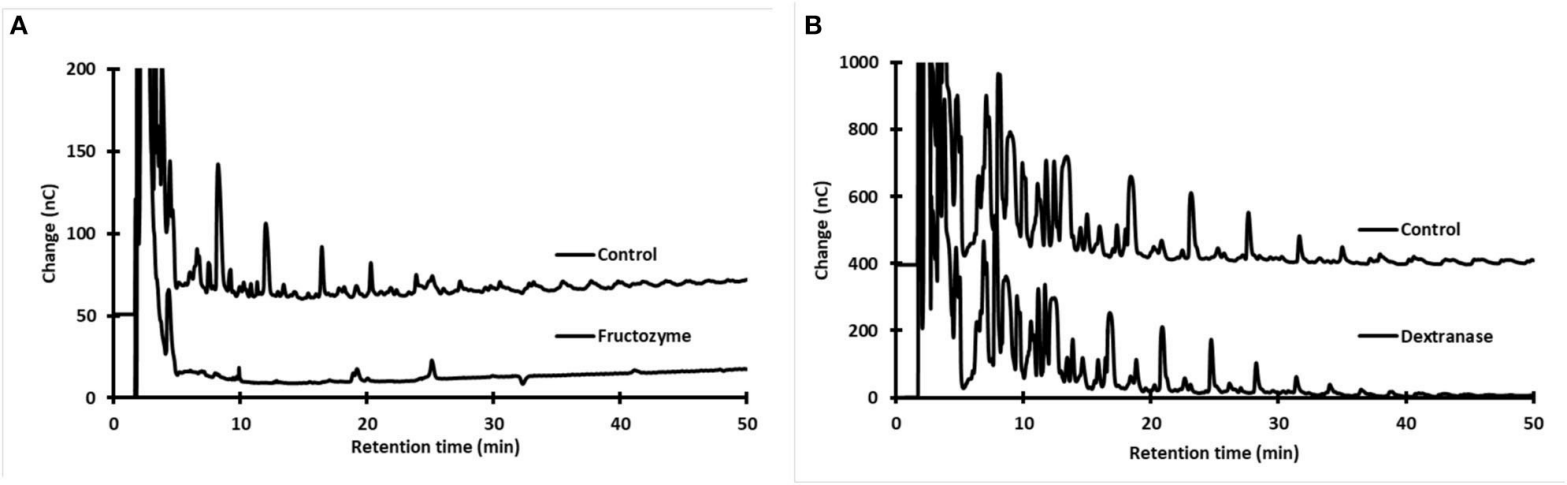
HPAEC-PAD oligosaccharide profile in samples of accumulated *aguamiel* before and after enzymatic treatment to identify the chemical nature of oligosaccharides. **(A)** Treatment with Fuctozyme. **(B)** Treatment with Dextranase. The analyses correspond to samples of accumulated *aguamiel* from plants P1 and P2 (*A. mapisaga*).

The dextranase treatment did not change the oligosaccharide profile (Figure 4B). In contrast, almost all the complex OS were hydrolyzed after the Fructozyme treatment (Figure 4A). These results indicate that OS present in accumulated type-1 and type-2 *aguamiel* are fructo-oligosaccharides with either β2-1 or β2-6 structure, but not an isomalto-oligosaccharide, α1-6-type structure, as suggested by the common presence of dextran in fermented *aguamiel*.

These results demonstrate the important activity of lactic acid bacteria that initiate the fermentation of *aguamiel* in the plant, synthesizing complex sugars (mainly fructo-oligosaccharides) from sucrose, as observed in type-1 and type-2 *aguamiel*. These sugars are produced from glucose released from sucrose, which also accounts for the contents of reducing sugars in accumulated *aguamiel*.

#### Microbial Influence on *Aguamiel* Changes During Accumulation

As pointed out by other authors (20, 28), one of the main challenges in controlling *aguamiel* fermentation for *pulque* production is the microbial definition of the process, and the fact that fermentation frequently starts during the accumulation of *aguamiel* in the plant. This is a very complex issue, as the traditional process requires human intervention both in the scraping process and during *aguamiel* collection. Modern plantations have reduced human intervention by using manual pumps, but most *pulque* production sites still use the traditional scraping and collection procedures. In addition, Martinez-Rodríguez et al. (22) identified an endogenous microbiota that could modify *aguamiel* during accumulation. This is consistent with the differences in composition observed between freshly collected and accumulated *aguamiel*. We therefore examined the microbiota in a sample of *aguamiel* collected immediately after scraping from one of our study plants in an early production stage, through metagenomic sequencing.

Table 3 shows that *Leuconostoc* is the most abundant genus of bacteria in fresh *aguamiel*; along with *Zymomonas*, these are also the essential microbial components of *pulque* fermentation. The two genera account for 82% of the microbiota in fresh *aguamiel*, being frequently reported in accumulated *aguamiel* (13, 29). Interestingly, these two genera are also abundant in *pulque* after 24 h fermentation but they account only for 44% of the organisms present in the microbial community (30), less abundant than in the fresh *aguamiel*, as found in this work. Moreover, *Zymomonas mobilis* is deemed essential for *pulque* production (11, 31) 36.1% of the species identified in the *aguamiel* sample correspond to *Z. mobilis*, previously reported in the *pulque* microbiota and already reported as equally important in *aguamiel* changes during the agave lifecycle (26). However, the total abundance of *Leuconostoc mesenteroides*, another species essential for *pulque* fermentation, was only 1.62% in our samples. Surprisingly, another unidentified *Leuconostoc* species accounted for 42.7% of bacterial abundance. Nevertheless, the synthesis of the additional fructo-oligosaccharides found in type-1 *aguamiel* may be the result of fructosyltransferases from *Leuconostoc* and *Zymomonas*, similar to the levan- and inulo-sucrases reported for *L. mesenteroides, L. paramesenteroides, L. citreum, L.kimchii*, and *L. plantarum* (32–36), or the levansucrases from *Z. mobilis*. These bacteria, isolated from fresh *aguamiel* immediately after scraping, are also the most abundant ones in accumulated *aguamiel* and play a central role in *pulque* fermentation, where *Z. mobilis* produces most of the ethanol while *Leuconostoc* species account for most of the pre- and probiotic compounds. Other bacteria species found in low abundance in *aguamiel* might rather be associated with the plant and have little influence on the fermentation process. Further research is needed to understand their role.

**Table 3:** Genera present in the microbiota of *fresh aguamiel* from *A. salmiana*.

**Domain**	**Genus**	**Number**	**%**
B	*Leuconostoc*	122,187	46.08
B	*Zymomonas*	95,398	35.98
B	*Acetobacter*	13,262	5.00
B	*Lactococcus*	12,396	4.67
B	*Acinetobacter*	8,527	3.22
B	*Gluconacetobacter*	3,848	1.45
B	*Bartonella*	3,415	1.29
V	*Punalikevirus*	1,500	0.57
B	*Lactobacillus*	1,246	0.47
B	*Pseudomonas*	1,005	0.38
E	*Saccharomycetaceae_unclassified*	405	0.15
E	*Debaryomycetaceae_unclassified*	304	0.11
B	*Pediococcus*	287	0.11
B	*Yersinia*	261	0.10
E	*Eremothecium*	257	0.10
B	*Gluconobacter*	243	0.09
B	*Gallionellaceae_unclassified*	194	0.07
B	*Parabacteroides*	126	0.05
B	*Pantoea*	80	0.03
B	*Providencia*	66	0.02
B	*Candidatus_Phytoplasma*	59	0.02
E	*Naumovozyma*	49	0.02
B	*Hahellaceae_unclassified*	42	0.02
B	*Hafnia*	4	0.00
	Total number	265,161	

*B, Bacteria; E, Eukarya; V, Viruses*.

### Changes in Sugar Concentration in *Aguamiel* and Scraped Tissue During the Productive Lifetime of Agave Plants

#### Sucrose and Reducing Sugars

Although the concentration of sugars in both fresh *aguamiel* and scraped tissue would be expected to change over the plant production lifetime, neither sugar composition nor its changes have been reported so far. The ANOVAs showed that sugar concentration in *aguamiel* collected after scraping and in the scraped tissue varies significantly over the several months of the plant productive lifetime.

**Figure 5:**
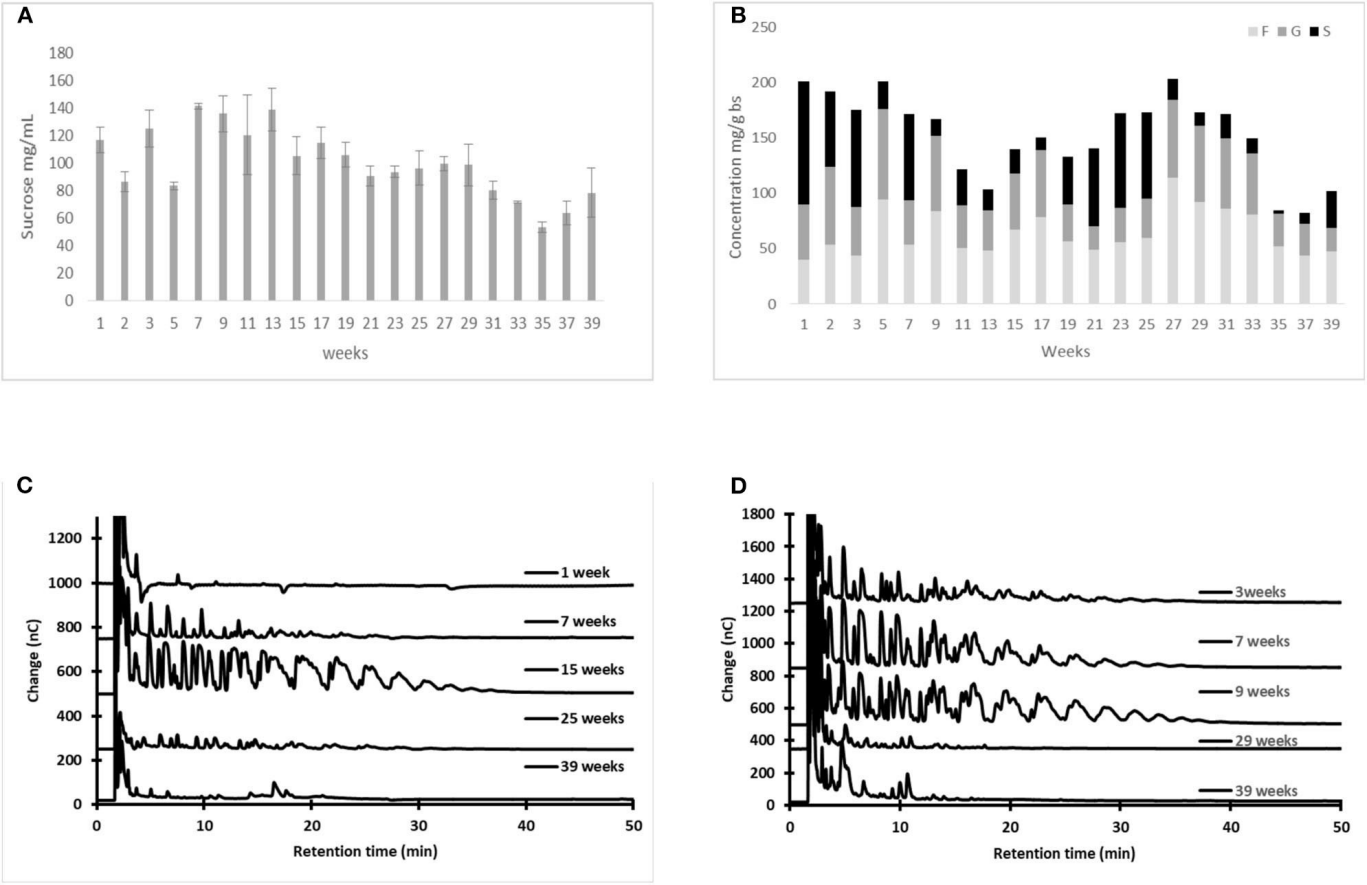
Changes in sucrose and fructan concentration, as well as in the FOS profile, in fresh *aguamiel* collected after scraping [**(A,C)**, respectively] and *metzal* [**(B,D)**, respectively] during the *agave* productive lifetime. Statistical differences: *p*-values for sucrose concentration (P1, *p* < 0.0005; P2 *p* < 0.0005; and P3 *p* < 0.0005), while for fructan concentration (P1, *p* < 0.0005; P2 *p* < 0.0005; and P3 *p* < 0.0005).

Sucrose concentration in *aguamiel* collected after scraping increased with time from 54, 99.2 to 123 g L^−1^ in the earliest collected samples of the three plants, to reach a peak level after several weeks of production (147, 125 and 134 g L^−1^, respectively). Although the peak sucrose concentration varied across plants, in all cases it was reached in the first two months of production and decreased thereafter until the end of the agave productive lifetime (Figure 5).

We believe that this behavior in *aguamiel* is a consequence of the stress caused to the plant by the “castration” procedure followed by the physical damage inflicted with daily scraping. In response, the plant carries out photosynthesis intensively to supply sucrose to the nutrient-deprived, damaged organs. The increasing sucrose content in *aguamiel* may also be a consequence of carbohydrate mobilization.

When, after several months, the agave plant reaches the latest production stage, the leaves nearest the *cajete* are removed to provide easier access to *aguamiel*. The removal of the remaining photosynthetic organs causes sucrose content in *aguamiel* to decrease. In the final stage of *aguamiel* production, the agave leaves, weakened by the systematic physical damage and the draining of their energy source, reach senescence (see Supplementary Material). These data confirm the centuries-old traditional knowledge: *agaves* are a source of sucrose, *aguamiel* literally means “honey-water.”

Unlike *aguamiel*, extracts of grinded scraped tissue contain glucose and fructose, besides sucrose. The proportion of reducing sugars increases as production time proceeds, undoubtedly as a result from invertase and/or Fructan exohydrolase (FEH) activity. Large amounts of simple sugars were observed in *metzal* between weeks 5 and 7.

#### Fructan Concentration and Degree of Polymerization

The highest concentration of fructans in fresh *aguamiel* was recorded between the first third and the midpoint of the productive lifetime, with the maximum amount of FOS recorded between weeks 9 and 15 (Figure 5C). Similarly, the maximum amount of FOS in tissue scraped from the three plants studied was recorded in samples obtained at weeks 7 and 15 of the *agave* productive lifetime (Figure 5D).

**Figure 6:**
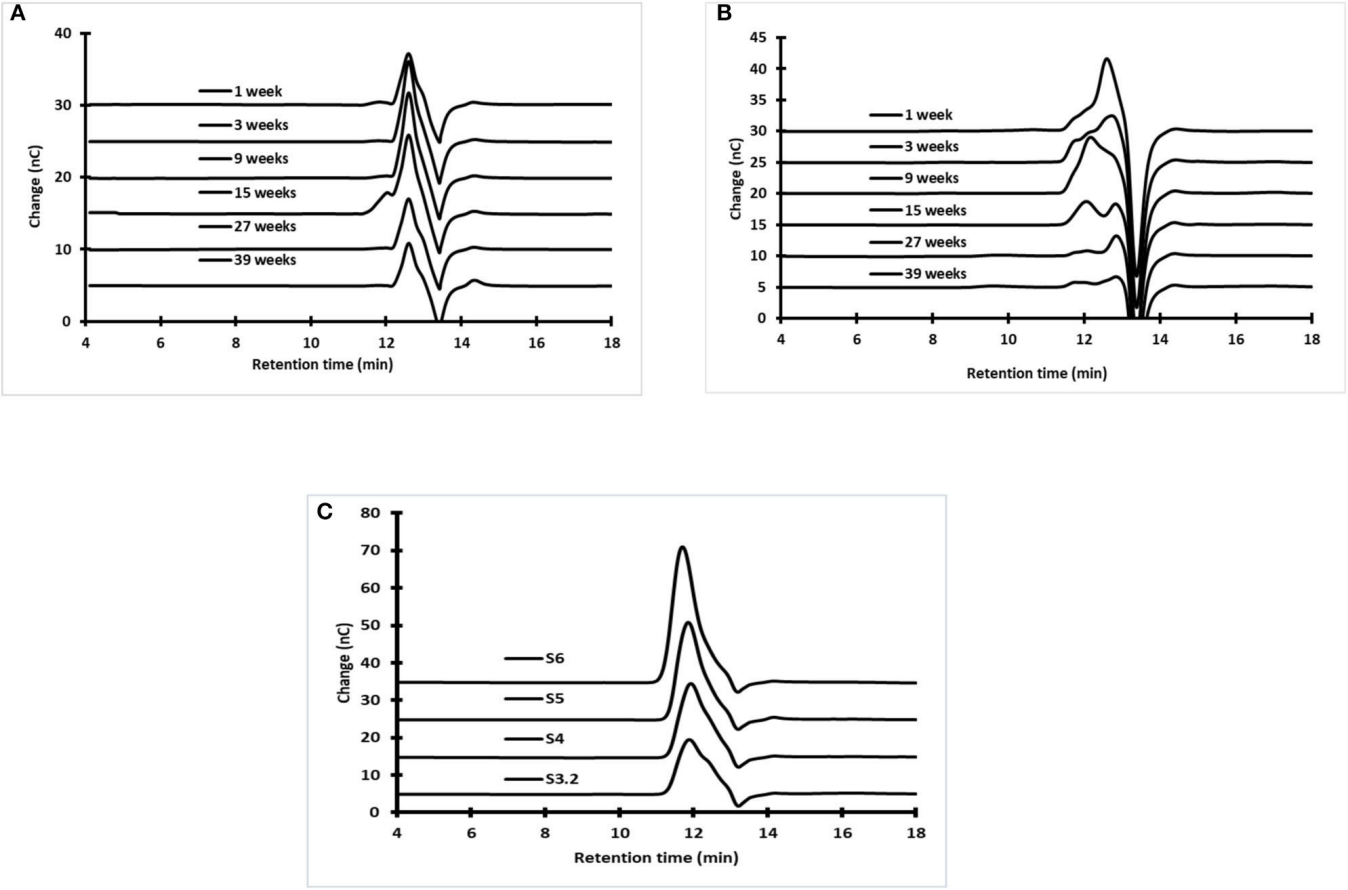
Fructan molecular weight distribution as determined by Gel Permeation Chromatography (GPC) in **(A)** fresh *aguamiel* and **(B)** *metzal* during the agave productive lifetime as well as in **(C)** Stem sections of reference plant (S3.2 = Upper stem section; S4 = section 4; S5= section 5 and S6 = lower stem section).

Figure 6 shows that, as determined by GPC, the normal molar mass distribution of fructans found in fresh *aguamiel* harvested from the three study plants varied over the productive lifetime. Table 4 summarizes the average molar mass in terms of weight (Mw) and number (Mn), and the polydispersity index of *aguamiel* from the three plants, as well as their changes over time (production weeks). The largest Mn and Mw values were observed at week 15 in P1, week 17 in P3, and at the last production week in P2. Thus, the time needed to reach the highest polymer molar mass in number and weight varies between plants. By comparing DPn and DPw, we concluded that fructan composition of *aguamiel* is heterogeneous (PI > 1), with the polydispersity index varying over the production season. Fructan*s* are less disperse at the beginning of the agave productive lifetime and become poly-disperse as the plant is exploited. This transition takes place between the first third and the midpoint of the productive lifetime, but varies between plants.

**Table 4:** Degree of fructan polymerization in fresh *aguamiel* collected after scraping and *metzal* from agave plants P1 and P2: *A. mapisaga*, P3: *A. salmiana*, during their productive lifetime.

	**Weeks**	**Sample**	**PD**	**Mn_**I**_**	**Mw_**I**_**	**Dpn_**I**_**	**Dpw_**I**_**	**Mn_**II**_**	**Mw_**II**_**	**DPn_**II**_**	**DPw_**II**_**
P1	3	A	1.1	322	366	2	2	–	–	–	–
		M	1.9	741	1,432	5	9	–	–	–	–
	7	A	1.1	324	367	2	2	–	–	–	–
		M	1.5	772	1,183	5	7	–	–	–	–
	9	A	1.9	556	1,050	3	6	–	–	–	–
		M	2.1	1,202	1,502	7	9	218	240	1	1
	31	A	2.0	415	822	2	5	–	–	–	–
		M	NA	1,296	1,605	8	10	218	248	1	1
	39	A	1.8	428	818	3	6	–	–	–	–
P2	3	A	1.1	338	382	2	2	–	–	–	–
		M	NA	1,139	1,542	7	9	218	240	1	1
	5	A	1.1	340	385	2	2	–	–	–	–
		M	NA	1,414	1,734	9	11	312	363	2	2
	9	A	1.1	342	389	2	2	–	–	–	–
		M	NA	1,072	1,428	7	9	230	254	1	1
	17	A	1.5	381	554	2	3	–	–	–	–
		M	NA	1,189	1,548	7	9	219	242	1	1
	27	A	1.9	439	842	3	5	–	–	–	–
P3	3	A	1.1	344	391	2	2	–	–	–	–
		M	1.9	641	1,208	4	7	–	–	–	–
	5	A	1.1	343	390	2	2	–	–	–	–
		M	1.8	705	1,301	4	8	–	–	–	–
	9	A	1.5	388	568	2	4	–	–	–	–
		M	NA	913	1,308	6	8	173	177	1	1
	17	A	1.8	435	762	3	5	–	–	–	–
		M	1.9	647	1,222	4	7	–	–	–	–
	23	A	1.6	366	580	2	4	–	–	–	–

*A: Fresh aguamiel, M: Metzal; NA: not applicable*.

The highest degree of polymerization by mass (DPw) of fructan in fresh *aguamiel* was recorded after 15 weeks in P1 (2.2), 27 weeks in P2 (2.6), and 17 weeks in P3 (2.6) with a DPw of 6.1, 5.1, and 4.6, respectively. Based on the degree of polymerization, we can conclude that FOS are the predominant fructans in *aguamiel*; this is consistent with what Ortiz-Basurto et al. (5) reported. The changes in the degree of polymerization of fructan during the production lifetime seem to be associated to plant stress, as discussed below.

#### Scraped Tissue

Little attention has been paid so far to the scraped tissue, *metzal*, an abundant residue of *aguamiel* production. Escobedo-García et al. (37) recently proposed its use as a functional ingredient in supplemented cookies. However, as described here, the production stage at which bagasse is collected should be carefully considered for any application. Figure 6 shows that a normal fructan Mw distribution is never attained in the scraped tissue, as observed in the reference plant stem (P4). Instead, the bimodal distribution denotes structural changes associated with production stress. Interestingly, except for the intermediate collections, fructans in *aguamiel* did attain a normal Mw distribution.

Fructans found in scraped tissue have a higher molecular weight than those in *aguamiel*. The largest fructan molecules found in scraped tissue had DPn = 9 and DPw = 11, whereas the largest polymers found in *aguamiel* had DPn = 3 and DPw=6. These differences in the degree of polymerization and molecular weight between *aguamiel* and scraped tissue further confirm our previous hypothesis that fructans in *aguamiel* come primarily from the apoplast and not from vacuoles, where larger fructans are stored.

### Sugar Concentration and Degree of Polymerization in the Agave *Pine*

Establishing a direct association between fructans in *aguamiel* and those in the plant is challenging. However, we examined fructan composition and distribution in a mature plant as a baseline for comparison vs. those in *aguamiel, metzal*, and in the pine. The core of the pine corresponds to the base of the *cogollo* and is cut off during agave “castration” to halt flowering and ensure that the carbohydrate reservoir of the plant is conserved for *aguamiel* production (38). The vegetative meristem and its surrounding tissue (young developing leaves) are also removed, leaving a cavity that serves as a container for *aguamiel* accumulation.

**Figure 7:**
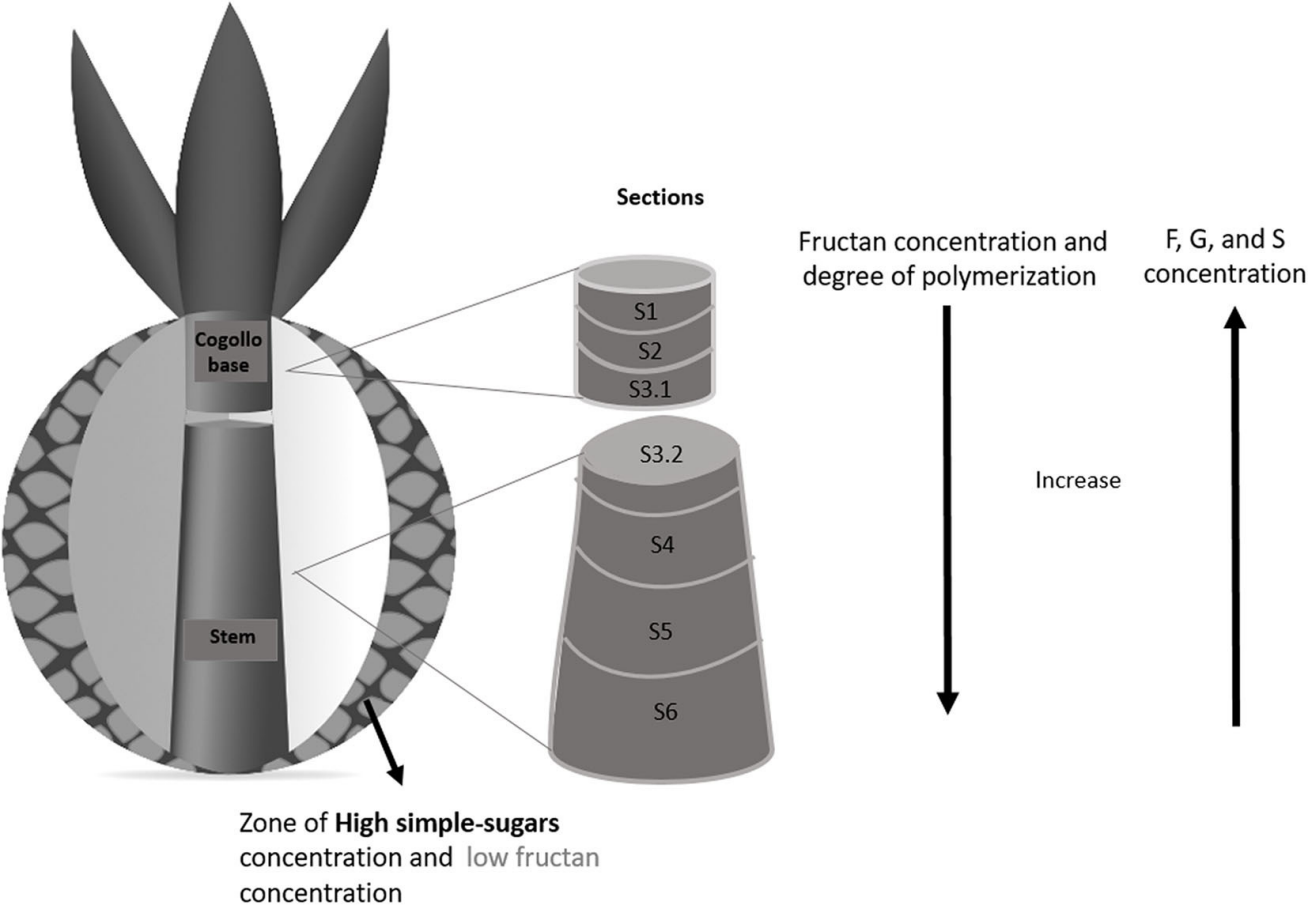
Schematics of the distribution of simple sugars and fructans in an *A*. *salmiana* pine. Fructan concentration of the center sample: S1:268.5, S2:260.3, S3.1:380.6, S3.2:307.8, S4:397.9, S5:531.6, S6: 565.9 mg/g of dry mass. Simple sugars concentration of the center sample S1:118.1, S2:67.9, S3.1:67.8, S3.2:39.9, S4:49.8, S5:28.5, S6:18.5 mg/g of dry mass.

Figure 7 shows the concentration of simple sugars and fructan in samples from different sections of the reference mature agave pine before castration. Simple sugars are present at the *cogollo* base, accounting for 20–50% of the total carbohydrate concentration in this section, while fructans account for the rest. Sugar contents varied between the different parts of the *cogollo* base. Concentration of simple sugars was lower in the core and increased toward the developing leaves, while fructan concentration showed the opposite pattern. As regards the radial distribution (see the cross section in Figure 7), the concentration of simple sugars was lower in the sections closer to the agave stem. The amount of fructans in the first two sections was similar but increased in the region closer to the agave stem.

No fructose or glucose was detected in the sections close to the base of the stem. Fructans constituted 307.8, 397.9, 531.6, and 565.9 mg/g of dry mass in the center of the sections S3.2, S4, S5, and S6, respectively (Figure 7), while fructose, glucose, and sucrose accounted for 39.9, and 49.8 mg/g of dry pine mass in the first two sections (S3.2, S4) of the stem. Finally, sucrose concentration in sections S5 and S6 was 28.5 and 18.5 mg/g dry mass.

These results are similar to those reported by García-Curbelo et al. (39) and were to be expected if we consider that the agave stem is a reservoir organ. The concentrations of fructose, glucose, and sucrose were lower in areas closer to the base of the stem, while fructan content increased. García-Curbelo et al. (39) also showed that the concentration and degree of fructan polymerization was higher in the region closer to the base of the stem, while simple sugars concentrate in the upper sections, away from the base. As described above, the fructan Mw distribution varied between pine sections. Figure 7 illustrates that in terms of DPn and DPw, that polymers are small in the first stem section and larger in the lower sections (DPn:13.4 and DPw:19.8), where a clear difference in degree of polymerization can be observed (see also Supplementary Material).

As explained in the previous section, the size and irregular (mainly bimodal) Mw distribution of fructans in scraped tissue depart from the normal distribution observed in the reference stem (Figure 7). These results demonstrate that these modifications involve fructan hydrolysis, as the degree of polymerization (in number and mass) in scraped tissue from an *aguamiel*-producing pine is considerably lower than in the intact stem.

As discussed previously, fructan size and concentration increase toward the base of the stem in the intact reference pine, while the lowest concentrations in the scraped tissue were recorded at the end of the plant productive lifetime, when the stem base is reached, suggesting fructan hydrolysis by FEH enzymes in the plant. Such hydrolysis is expected, given the huge stress to which the plant is subjected over the entire period of *aguamiel* production, particularly at the base of the stem where fructan concentration is higher. The fructose or FOS resulting from hydrolysis may then be transported to the apoplast through *cajete* cell walls to protect the plant from the damage produced by scraping and exposure to the environment. Based on the studies by Livingston and Henson (19), the hydrolysis and mobilization of fructan likely start with castration in order to fulfill an energetic role, avoid dryness, or induce an immune response (40), thus affecting the fructan profile and concentration in *metzal* and *aguamiel*.

### Total Sugar Yield During the Production Process

The total amount of sucrose produced over the entire agave production lifetime was estimated based on the assumption that sucrose concentration in *aguamiel* was the same in the two daily collections (24 h). This assumption is based on our results (see section Daily Changes in Aguamiel Composition above) showing that sucrose concentration remained almost constant from 7:00 to 14:00 h. This value is likely an overestimation, since plants are drained twice a day and CAM plants capture more CO_2_ during nighttime and increase gluconeogenesis during daytime (41). Similarly, the total amount of fructans produced during the entire agave production lifetime was estimated based on the assumption that the fructan concentration measured in the first 15 mL of *aguamiel* collected after scraping is the same for the first 200 mL. This assumption is based on our results (see section Fructan Dilution in *Aguamiel*) showing that fructans occur in the highest concentration in the initial volume and are still present, but in small amounts, after 200 mL have been collected. Thus, this is also an approximate figure.

Table 5 shows the estimates thus obtained. An estimated total of 70.6, 61.5, and 36.8 kg of sucrose and 3.0, 1.7, and 2.1 kg of fructans were obtained in 676.6, 662.3, and 334.9 L of *aguamiel*. This corresponds to an overall average of 9–11% w/v of sucrose, and 0.2–0.6% w/v of fructans in the *aguamiel* collected. Concentration of simple sugars in scraped tissue are higher than in the intact plant, strengthening the hypothesis that the plants respond to stress by hydrolyzing fructans, which are then recovered in *aguamiel*.

**Table 5:** Estimated total amount of sugars obtained from *aguamiel* and *metzal* over the entire productive lifetime of agave plants: P1 and P2: *A. mapisaga and* P3: *A. salmiana*.

		**P1**	**P2**	**P3**
Aguamiel	Volume (L)	676.6	662.3	334.9
	Sucrose (Kg)	70.6	61.5	36.8
	Fructans (Kg)	3.0	1.7	2.1
Metzal	Mass (Kg)	37.2	21.5	23.5
	FGS (Kg)	0.9	0.5	0.6
	Fructans (Kg)	1.3	0.4	0.9
	% w/w^*^	6.0	4.0	6.4

FGS: Fructose, glucose y sucrose;

* *wet weight (Fructans + FGS/Total wet mass) Nota 2. The P4 stem was a wet weight of 8.2 Kg, 0.1 Kg of FGS and 1.3 Kg of Fructans*.

The estimated total amount of sugars (fructose, glucose, sucrose, and fructans) in scraped tissue ranged between 4 and 6% w/w of total wet mass; total carbohydrates in the intact pine accounted for 17% w/w. According to our estimates, the amount of fructans lost in the scraped tissue represents 1.3, 0.5, and 0.9 kg, or 30%, 22.7%, and 30% of the total amount originally in the pine. The amount of fructans lost in scraped tissue represents a significant waste in the standard *aguamiel* production process, which has been rarely considered so far.

## Concluding Remarks

We examined the composition of soluble carbohydrates, especially fructans, during the entire process of *aguamiel* harvesting. We believe that this new information would warrant a detailed revision of the procedures used for producing *aguamiel* and its final product (*pulque*) in order to increase the availability of fructans, one of the richest nutriments in agave. We showed that *cajete* scraping is the main source of the fructans found in *aguamiel*. Our results could inform the design and adoption of improved harvesting practices that yield higher concentrations of fructans in *aguamiel* as well as innovative practices for recovering the huge amount of fructans lost in the scraped tissue (*metzal*). We also found that the taxonomic profile of the fresh aguamiel compared to the *pulque* microbiota differed mainly in the abundance of the *Leuconostoc* genus which species might play an important role in defining the different carbohydrate profiles in aguamiel.

## Data Availability Statement

The raw data supporting the conclusions of this article will be made available by the authors, without undue reservation.

## Author Contributions

AL research lider, designed the experiments and gave general supervision, and wrote the manuscript. IP-G MSc student, did almost all the experimental work, and wrote the initial version of the manuscript. FG-M gave technical support in all areas, including field experiments, and developed techniques to measure oligosaccharides and sugars. R-AME gave technical support in molecular biology and analytical techniques, including enzymatic analysis, and DNA extraction. AS-F did all work related with aguamiel microbiota: DNA sequencing, data analysis, and discuss the results with AL. All authors contributed to the article and approved the submitted version.

## Conflict of Interest

The authors declare that the research was conducted in the absence of any commercial or financial relationships that could be construed as a potential conflict of interest.
