# Supplementary material for: Evolution of Fructans in Aguamiel (Agave Sap) During the Plant Production Lifetime
Source: Front Nutr. 2020 Oct 8;7:566950. doi: 10.3389/fnut.2020.566950 (PMC7581979; doi:10.3389/fnut.2020.566950)
Supplement: Supplementary file 1 [file Data_Sheet_1.docx]

**Supplementary material**

Tabla 1S. Fructans polymerization degree from central zone of different sections (S3.2, S4, S5, S6) of P4 stem (*A. salmiana*).

|  | **Section** | **Mn** | **Mw** | **PI** | **DPn** |  | **DPw** |
| --- | --- | --- | --- | --- | --- | --- | --- |
| **Stem** | S3.2 | 1453.2 | 2285.4 | 1.6 | 8.9 |  | 13.9 |
|  | S4 | 1366.9 | 2078.8 | 1.5 | 8.3 |  | 12.7 |
|  | S5 | 1583.1 | 2299.4 | 1.5 | 9.6 |  | 14.1 |
|  | S6 | 2187.4 | 3236.6 | 1.5 | 13.4 |  | 19.8 |

1. P3
2. P2
3. P4
4. P1


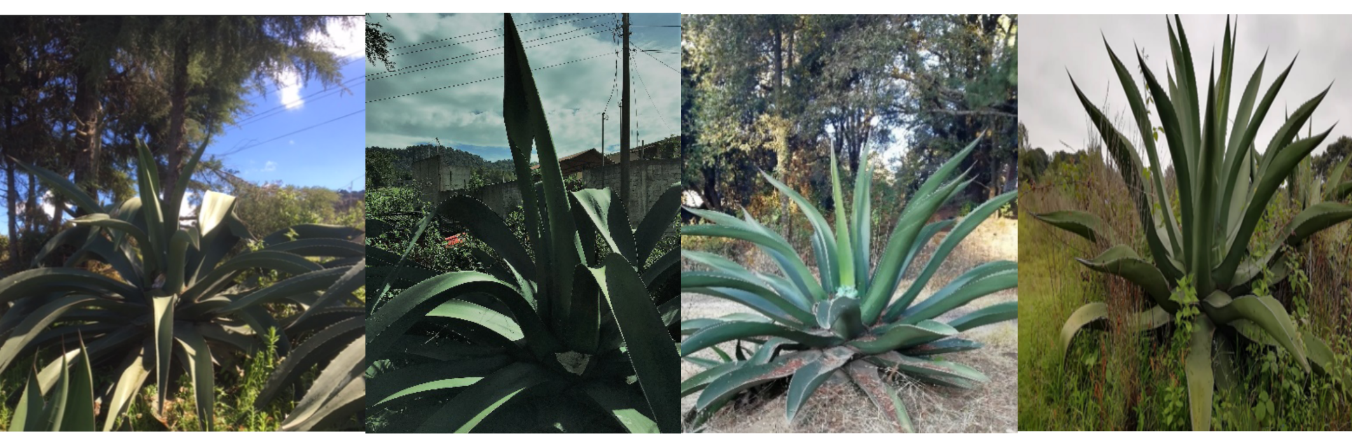


Figure 1S. Plant used for this study: a) P1 *A. mapisaga*, b) P2 *A. mapisaga, c)* P3 *A. salmiana y* d) P4 *A. salmiana*

**
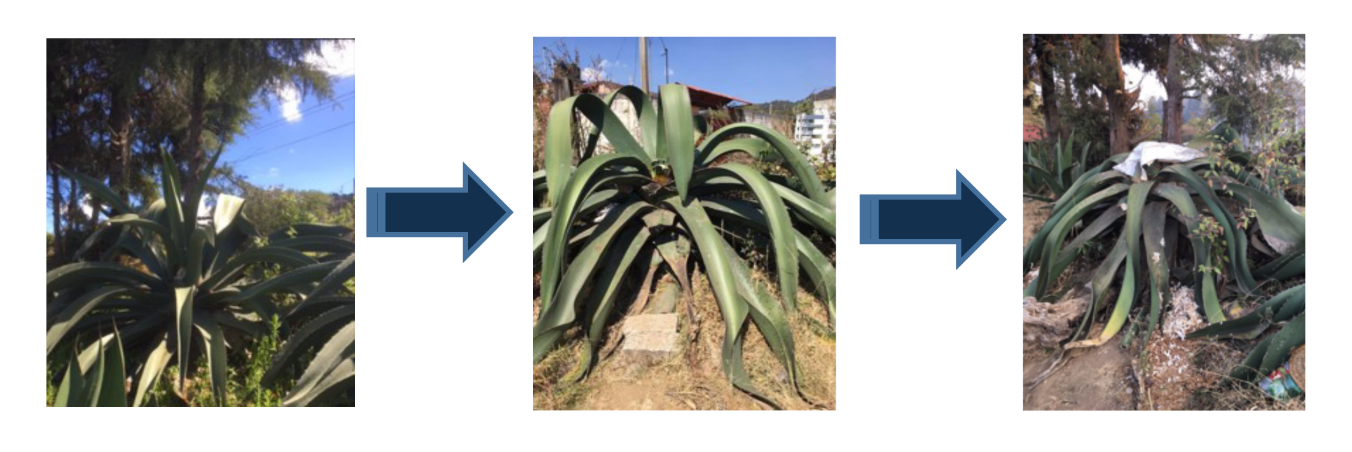
**

Figure 2S. P1 (*A. mapisaga*) during *aguamiel* production lifecycle.

c)

a)

b)


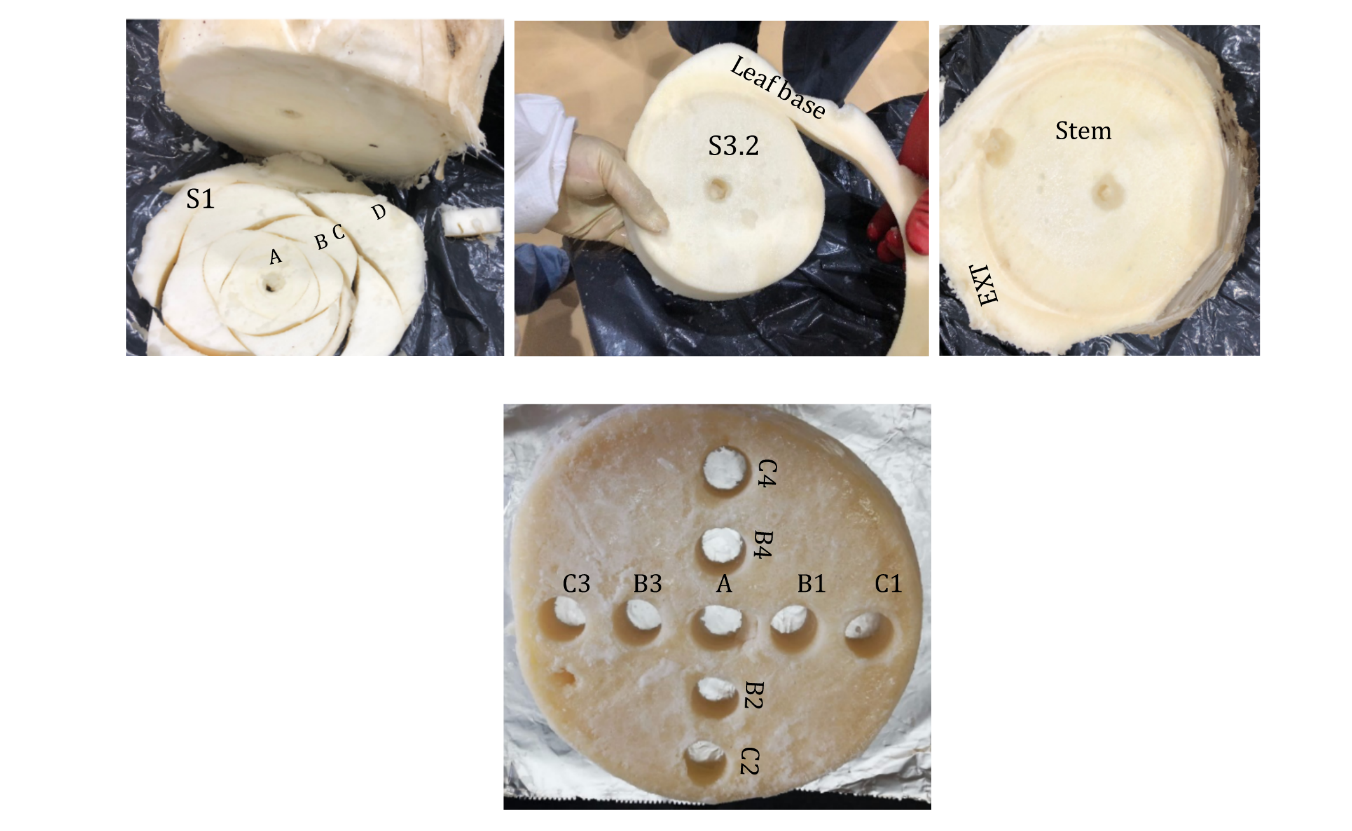


d)

Figure 3S. Sampling of P4 (*A. salmiana*): a) 1^st^ section, cogollo base, b) 4^th^ section, apex stem , c) stem exposed from pine, d) samples taken from 4 section.


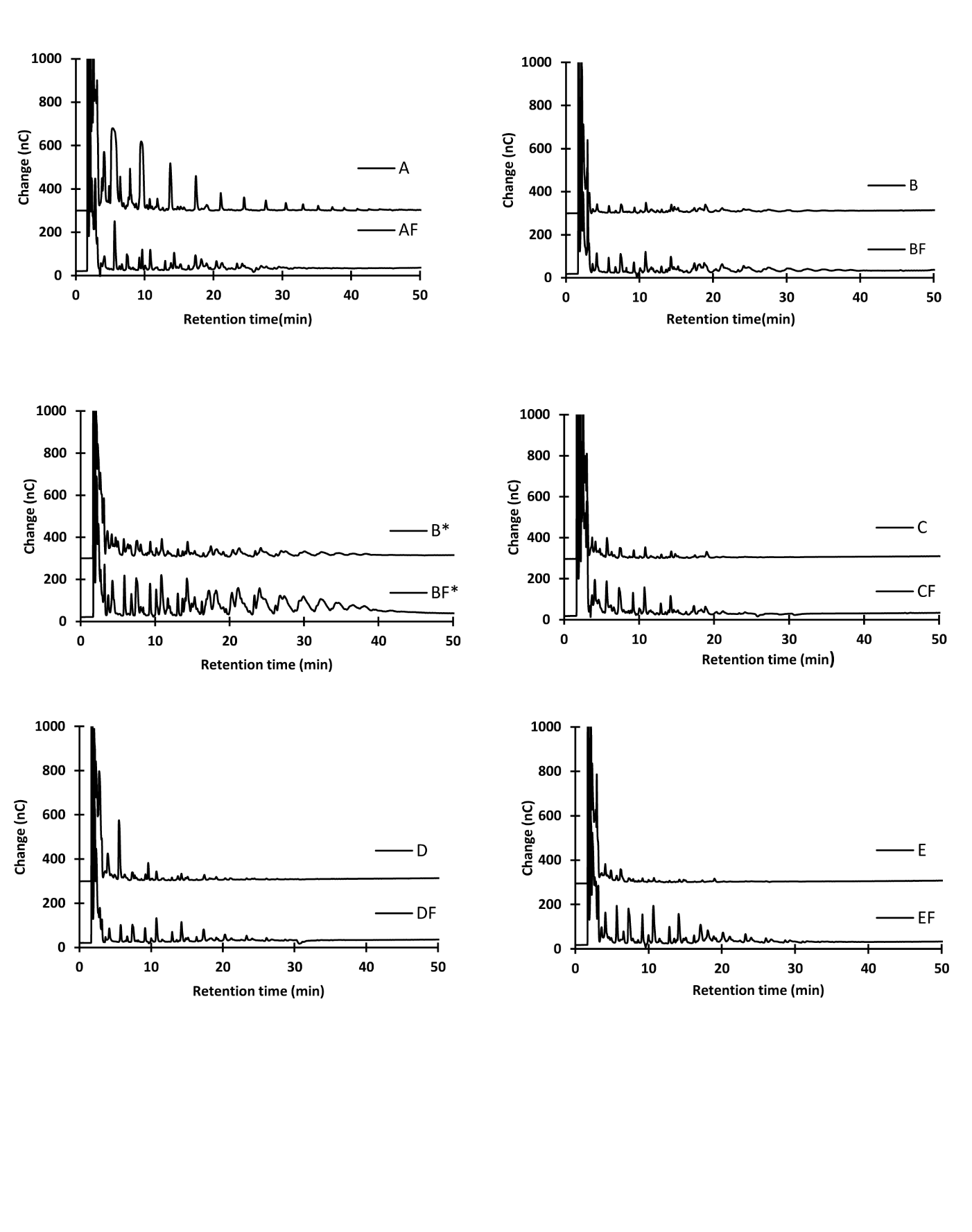


b) Type 3

1. Type 1

a)

c) Type 2

d) Type 3

f) Type 2

e) Type 2


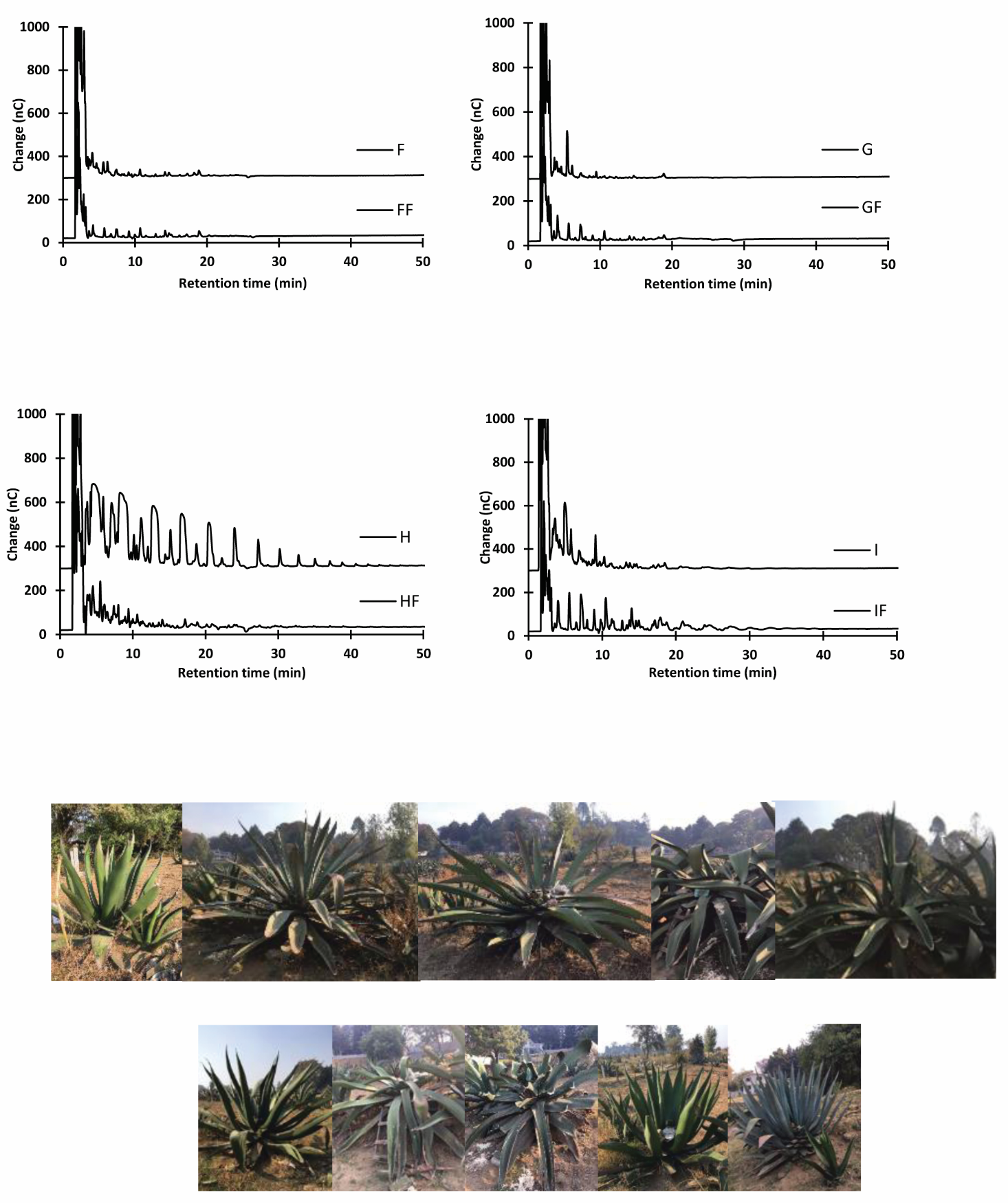


e) Type 2

f) Type 2

h) Type 2

g) Type 1

D

C

B*

B

A

I

H

G

F

E

Figure 4S. **Examples of the three different FOS profiles observed in accumulated *aguamiel* from 10 extra plants**. HPAEC-PAD oligosaccharide profiles in fresh (immediately after scrapping) and accumulated (10 h) *aguamiel*.
